# Anaesthesia in PROstate Biopsy Pain Obstruction Study: A Study Protocol for a Multicentre Randomised Controlled Study Evaluating the Efficacy of Perineal Nerve Block in Controlling Pain in Patients Undergoing Transperineal Prostate Biopsy

**DOI:** 10.3389/fsurg.2021.649822

**Published:** 2021-10-06

**Authors:** Bi-Ming He, Rong-Bing Li, Hai-Feng Wang

**Affiliations:** Department of Urology, Shanghai East Hospital, School of Medicine, Tongji University, Shanghai, China

**Keywords:** prostate biopsy, transperineal, perineal nerve block, randomised controlled trial (RCT), pain

## Abstract

**Introduction:** Transperineal prostate biopsy is as effective as the transrectal biopsy in detecting prostate cancer and has a lower risk of infection. However, concerning the procedural pain of the transperineal route, a higher level of anaesthesia is needed, which prevents this approach from being widely used. Although several methods of local anaesthesia to relieve pain during transperineal biopsy have been described, few well-designed trials have been conducted to assess the efficacy of local anaesthesia.

**Methods:** This is a prospective, multicentre, randomised controlled study in men suspected of having prostate cancer and planning to undergo transperineal prostate biopsy. The aim of this trial is to determine whether the perineal nerve block and periprostatic block relieve pain to different extents in men undergoing transperineal biopsy. The main inclusion criteria are men aged between 18 and 80 years old, a prostate-specific antigen (PSA) level of 4–20 ng/ml, or/and suspicious rectal examination findings. A sample size of 190 participants, accounting for a 10% loss, is required. All participants will be randomly allocated at a ratio of 1:1 to the perineal nerve block (*n* = 95) and periprostatic block groups (*n* = 95). The primary outcome will be the level of the worst pain experienced during the transperineal prostate biopsy procedure, which will be measured by a numerical rating scale (NRS). The key secondary outcomes will include the pain severity score at 1, 6, and 24 h after prostate biopsy.

**Results:** The primary outcome is the level of the worst pain experienced during the prostate biopsy procedure. The main secondary outcomes are as follows: (1) Post-biopsy pain severity score at 1, 6, and 24 h after the prostate biopsy; (2) Changes in blood pressure, heart rate and breathing rate during the biopsy procedure; (3) External manifestations of pain during biopsy; (4) Anaesthesia satisfaction; (5) The detection rate for clinically significant prostate cancer and any prostate cancer.

**Conclusion:** Anaesthesia in PROstate biopsy Pain Obstruction Study (APROPOS) is randomised controlled trial aiming to determine the efficacy of the perineal nerve block in controlling pain in patients undergoing prostate biopsy *via* the transperineal approach.

**Clinical Trial Registration:** www.ClinicalTrials.gov, identifier: NCT04501055.

## Introduction

Prostate cancer (PCa) is the second most frequently diagnosed malignancy and the fifth leading cause of death among males worldwide (1). Men with an elevated serum prostate-specific antigen (PSA) level and abnormal findings on digital rectal examinations (DREs) or transrectal ultrasonography (TRUS) examinations are usually suspected of having of prostate cancer. In the clinic, males who are suspected of having PCa typically undergo a prostate biopsy to obtain specimens for pathological diagnosis.

Prostate biopsies are mainly performed by either the transrectal or transperineal approach. Although these two methods differ little in the overall cancer detection rate (2, 3), the transperineal approach has a lower incidence of infection because the instrument is inserted from the perineum to the prostate, which avoiding damage to the rectal wall (4).

While the transperineal approach has merits, the severe pain caused by this approach has prevented its widespread use for prostate biopsies. Compared with the transrectal approach, thetransperineal approach causes more pain (5). Hence, unlike the transrectal approach, which can be performed under local anaesthesia only, transperineal biopsies require general anaesthesia in some cases (6), which takes more time, is costlier, and is associated with more anaesthesia-related risks. To date, several local anaesthesia methods for transperineal biopsy have been described (7–10). The periprostatic block procedure is the recommended and accepted method for patients undergoing a transperineal biopsy (11), although it was first described for use for the transrectal approach (12).

We describe a local anaesthesia method, the perineal nerve block, to reduce the procedural pain of transperineal biopsies; the method was developed on the basis of an anatomical study, and then a single-centre randomizedtrial was conducted to preliminarily verify its efficacy and safety (13). This method showed a reasonable positive effect on pain measured using a visual analogue scale (VAS). Hence, we plan to conduct this multicentre randomised controlled trial to confirm the findings and ensure the results are generalizable.

The aim of this trial is to compare the perineal nerve block and periprostatic block in terms of pain control in patients undergoing a prostate biopsy *via* the transperineal approach. The primary objective is to assess whether the perineal nerve block is superior to the periprostatic block in relieving the procedural pain related to transperineal biopsies.

## Trial Design

In this prospective, multicentre, randomised controlled trial, we anticipate to enrol 190 patients who are scheduled to undergo a transperineal prostate biopsy. The participants will be randomised to the perineal nerve block or periprostatic block groups.

**Figure 1:**
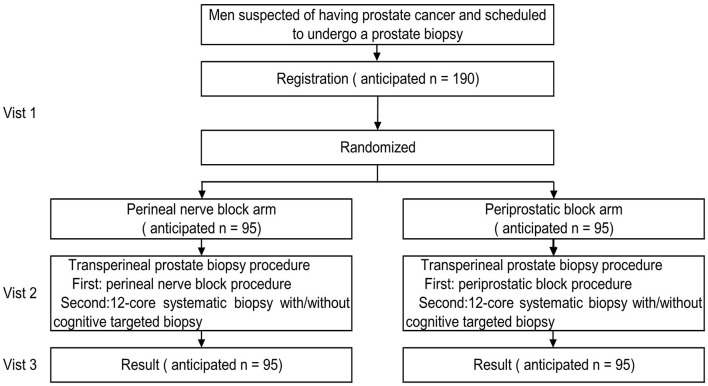
Trial flow chart.

The trial flow chart is shown in Figure 1, and the details and timeframe are shown in Table 1. The primary objective of this study is to assess the efficacy of the perineal nerve block compared with that of the periprostatic block in relieving pain.

**Table 1:** Timeline of the study protocol for participants.

**Contact with patient**								
	**Visit 1**	**Visit 2**						**Visit 3**
	**0**	**Pre-biopsy**	**Biopsy procedure**	**1-min post-biopsy**	**1-h post-biopsy**	**6 h post-biopsy**	**24 h post-biopsy**	**2 weeks post-biopsy**
Consent	×							
Screening	×							
Baseline characteristic	×							
PSA	×							
MRI	×							
Randomisation	×							
Perineal nerve block (Perineal nerve block arm)		×						
Periprostatic block (Periprostatic block arm)		×						
Transperineal prostate biopsy (12-core systematic biopsy with/without cognitive targeted biopsy)			×					
Blood presure measurement		×	×	×				
Heart rate measurement		×	×	×				
Breath rate measurement		×	×	×				
External manifestations assessment			×					
NRS pain score assessment				×	×	×	×	
Anaesthesia satisfaction assessment							×	
Pathological assessment								×
Withdrawal	Complete as required at any time following registration							
SAE	Complete as required at any time following registration							

## Outcomes

The primary outcome is the level of the worst pain experienced during the prostate biopsy procedure Pain will be measured by a numerical rating scale (NRS) ranging from 0 to 10, where 0 represents no pain and 10 represents the worst pain imaginable. Additionally, we will measure the pain experienced during the phlebotomizing procedure, with the pain experienced when the needle first touches the skin at the start of anaesthesia as the baseline or reference. Additionally, the adjusted NRS pain score (an adjusted score of 1 will be defined as the worst pain during the biopsy procedure minus the pain of phlebotomizing, and an adjusted of 2 will be defined as the worst pain during the biopsy procedure minus the pain of the needle first touching the skin at the start of anaesthesia) will be reported.

The main secondary outcomes are as follows:

▸ Post-biopsy pain severity score, for which pain will be evaluated by the NRS at 1, 6, and 24 h after the prostate biopsy.
▸ Changes in blood pressure, heart rate and breathing rate during the biopsy procedure, which will be measured and recorded by a multi-parameter monitor from 1 min prior to anaesthesia (initial value) to 1 min after the prostate biopsy. The change will be defined as the difference between the average value during the anaesthesia and biopsy procedures and the initial value. Additionally, the difference between the maximum value and the initial value will be assessed.
▸ External manifestations of pain during biopsy, which will be assessed by a research nurse who will be blinded to the block arm during the biopsy procedure. The pain assessment will be divided into five parts: the degree of facial expression (0 points for no particular expression or smile; 1 point for an occasional grimace or frown, a withdrawn expression, or a disinterested expression, 2 points for frequent or constant quivering chin or a clenched jaw), the degree of activity (0 points for lying quietly or being in a normal position, 1 point for slight contractions of the hip muscles or slight movements of hip, 2 points for severe contractions of the hip or lifting the hip out of the bed), the degree of voice expression (0 points for quiet or normal communication, 1 point for an occasional moan or weeping sound, 2 points for constant moaning or sobbing and screaming), the degree of pacification (0 points for being peaceful and not requiring pacification, 1 point for being able to be comforted easily, 2 points for being difficult to comfort) and the degree of cooperation (0 points for being calm and cooperative, 1 point for language resistance, 2 points for body resistance).
▸ Anaesthesia satisfaction, or patient satisfaction with overall anaesthesia, which will be measured by a questionnaire at 24 h after the biopsy. Five items will be included to evaluate satisfaction: whether the pain during the biopsy was less severe than expected (scores from 0 to 10, where 0 represents far less severe, and 10 represents far more severe than expected); whether the pain after anaesthesia was less severe than the pain during anaesthesia (scores from 0 to 10, where 0 represents far less severe and 10 represent far more severe); weather the patient is satisfied with the overall feeling of the biopsy (scores from 0 to 10, where 0 represents the highest level of satisfaction, and 10 represents the lowest level of satisfaction); whether the patient would recommend this type of biopsy to other patients (scores from 0 to 10, where 0 represents they would highly recommend, and 10 represents they would definitely not recommend it); and whether the patient would still want to choose this way if they have to undergo another biopsy (scores 0 to 10, where 0 represents very willing to choose it and 10 represents extreme reluctance).
▸ The detection rate for prostate cancer, defined as the proportion of men with prostate cancer among those who undergo a prostate biopsy.
▸ The detection rate for clinically significant prostate cancer, defined as the proportion of men with prostate cancer of International Society of Urological Pathology (ISUP) grade 2 or higher, according to the 2014 ISUP classification, among men who undergo a prostate biopsy.
▸ Adverse events, which will be recorded 7 days after this trial. These events mainly include haematuria, vagal reflex, infection, urinary retention and other adverse events identified by the Common Terminology Criteria for Adverse Events (CTCAE).

## Methods and Analysis

### Patient Population

Patients who fulfil all items of the inclusion criteria and exclusion criteria will be considered qualified to register for this trial. The inclusion criteria include an age between 18 and 80 years old, a PSA level of 4–20 ng/ml, and/or suspicious rectal examination findings. Volunteers will not be recruited if they have a history of an allergy to the study drug, symptomatic acute/chronic prostatitis or contraindications for a biopsy.

### Randomisation

The participants who meet the criteria and sign the consent form will be allocated at a ratio of 1:1 to the perineal nerve block arm or periprostatic block arm by using block randomisation. The random sequence will be generated by the PROC PLAN statement of the SAS program and then sealed in envelopes. The randomised number will be known and kept by one research nurse and blinded to the others. The randomised number will be revealed to the urologist (the one who will perform anaesthesia) only when the participant has already entered the operation room for anaesthesia and the prostate biopsy.

## Interventions

### Preparation Before the Block

All participants will be placed in the lithotomy position with a multi-parameter monitor. The participant's blood pressure, heart rate and breathing rate will be measured automatically every min, from 1 min before the anaesthesia procedure to 1 min after the prostate biopsy process. A urologist will be informed of which block method to perform on the spot after the research nurse open a sealed envelope.

### Perineal Nerve Block Arm

**Figure 2:**
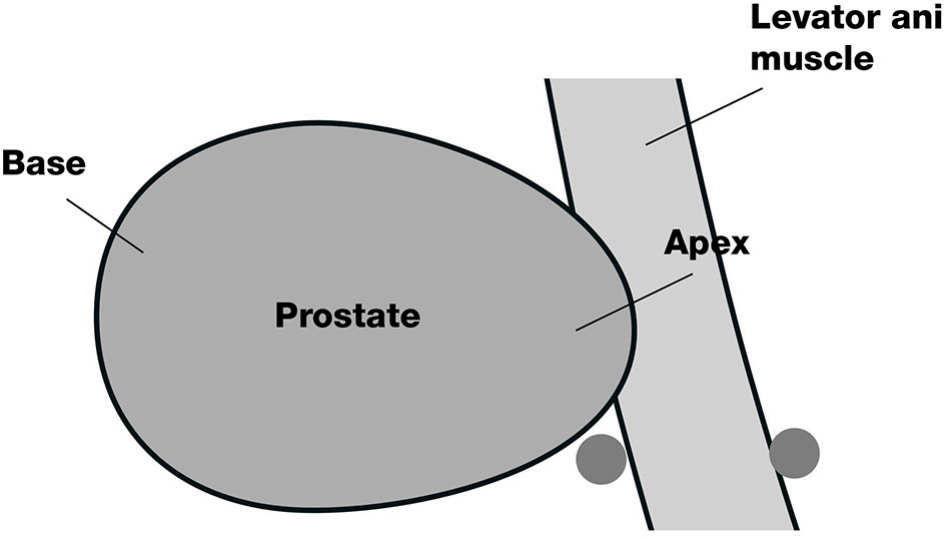
Diagram of the location of the perineal nerve block: The 2 grey dots are the block sites.

**Figure 3:**
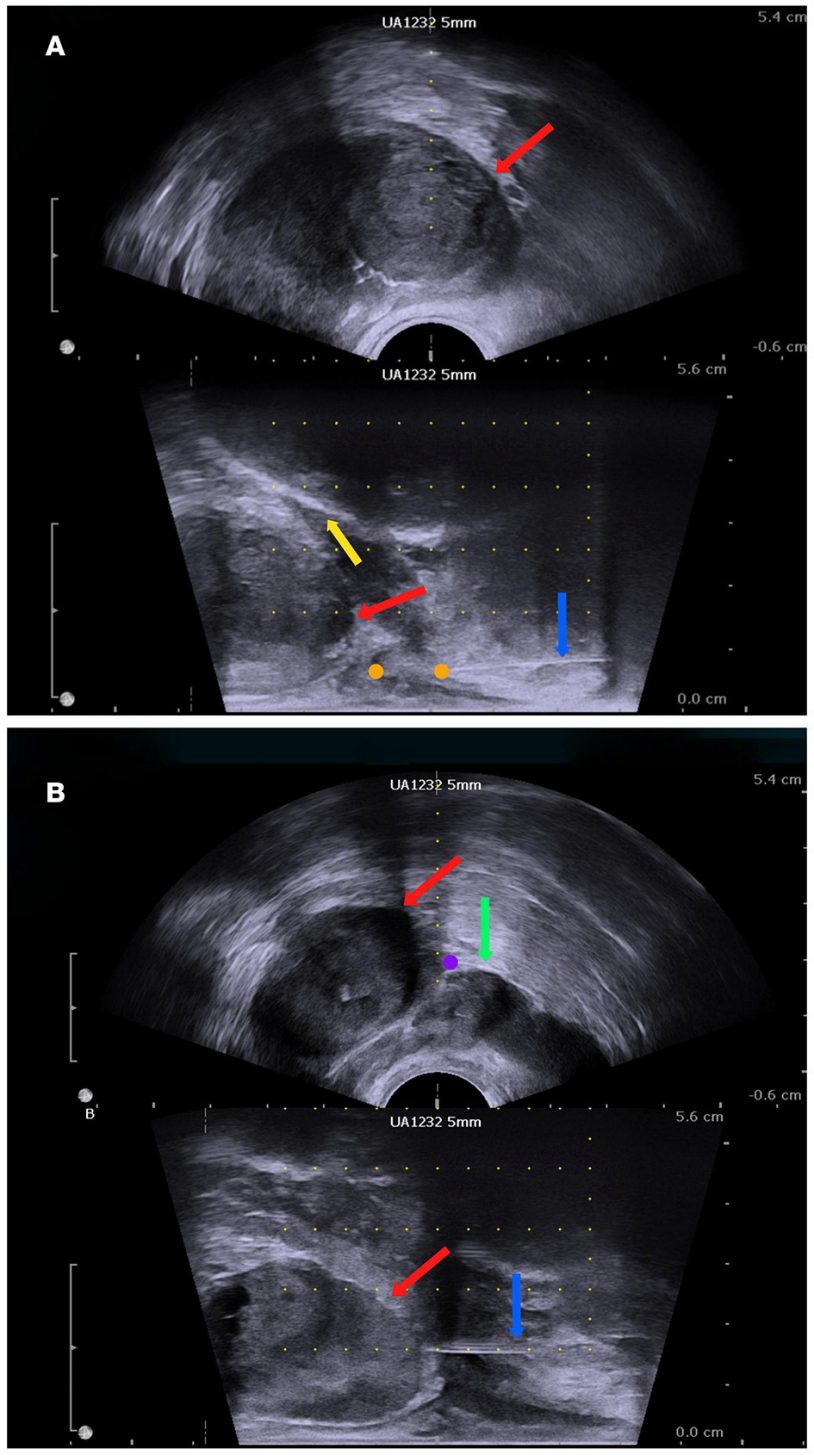
The biplanar transrectal ultrasound image. **(A)** Perineal nerve block. **(B)** Periprostatic block: prostate (red arrow); pubis (yellow arrow); seminal vesicle (green arrow); anaesthesia needle (blue arrow); perineal nerve block site (orange dot); periprostatic site (purple dot).

The participants in this arm will undergo a perineal nerve block before a prostate biopsy. The insertion site on the skin will be located on the horizontal line of the anal canal at the upper margin, 20 mm beside the midline. First, the insertion site will be anaesthetized with 10 ml of 1% lidocaine with a 22-gauge 32 mm needle. Then, an advanced injection of perineal nerve block will be carried out with a 20-gauge and 80 mm needle. We will inject 5 ml of 1% lidocaine at each site of the perineal nerve bundle under the guidance of a biplanar ultrasound transducer (Figures 2, 3A).

### Periprostatic Block Arm

In this arm, the periprostatic block procedure will be performed *via* an 80 mm 20-gauge needle under the guidance of the ultrasound probe after the skin is anaesthetised by 10 ml of 1% lidocaine *via* a 22-gauge 32 mm needle. The block region will be located on the basal prostatic capsule, lateral to the location between the prostate and seminal vesicle (Figure 3B). Additionally, 5 ml of 1% lidocaine will be injected at each site.

### Prostate Biopsy

Another urologist who is blinded to the block method will perform the prostate biopsy. All patients will undergo a 12-core systematic transperineal prostate biopsy with or without a targeted biopsy. The 12-core systematic biopsy region will be taken as described previously (14). A targeted biopsy will be performed using the cognitive fusion method (15), only when there is at least one suspicious lesion [defined as a lesion with a score ≥3 according to the Prostate Imaging Reporting and Data System (PI-RADS) criteria (16)] shown by the MRI. Another research nurse who is also blinded to allocation will record the manifestations of the patients during the biopsy procedure.

### Histology

The pathologic results will be reported within 14 days after the biopsy according to the ISUP guidelines (17). The pathologists who assess the biopsy samples will be blinded to all clinical data including the anaesthesia technique. The Gleason score, the length of the tumour, and the percentage of the tumour will be reported for each needle sample. Clinically significant cancer cases will be defined as those with a ISUP score of 2 or higher.

### Sample Size

The prospective data in our previous study showed a mean maximal VAS score of 1.8 for patients who underwent a biopsy with a perineal nerve block and a mean maximal VAS score of 3.4 for those who received a periprostatic block. The standard eviations for these two methods were 1.02 and 0.96, respectively.

For the calculation of sample size, using a power of 90%, a two-sided α of 5%, and an allocation ratio of 1:1 and assuming an NRS score for the perineal nerve block of 2.0, an NRS score for the periprostatic block of 2.5, and the standard deviations for these two methods are equal to 1.0, 86 men per arm will be required. Accounting for a withdrawal/loss rate of 10%, a total of 190 participants are required for inclusion.

### Statistical Analysis

The primary outcome in this trial will be analysed following the intention-to-treat principle as well as the per-protocol principle. The difference between the two groups will be evaluated with a 95% confidence interval (CI) using a generalised linear mixed model to express the precision of the estimate. In this model, the NRS pain score and the group will be considered the dependent variable, the imbalanced baseline characteristic will be considered the covariate, and the centre will be considered a random effect.

The secondary outcomes will be analysed with Pearson chi-square tests and expressed with 95% CIs. Each *P*-value reported in this trial will be two-sided.

### Harms and Adverse Events

There are few reports of severe adverse events (SAEs) regarding transperineal prostate biopsies and local anaesthesia for biopsies. The main expected adverse events or side effects are listed at Table 2, and the participants will be informed of the risk for these adverse events or side effects before registration. The expected incidence of the adverse events is based on both our previous data and the literature (18). All complications and adverse events that occur from the date of registration to 1 week after the biopsy will be recorded by a research nurse. SAEs are defined as any of the following: (1) death; (2) life-threatening; (3) hospitalisation; and (4) disability or permanent damage. SAEs will be recorded immediately and then sent to the ethics committee and the APROPOS monitoring board within 24 h.

**Table 2:** Other side effects that may occur in this trial besides pain.

**Side effect**	**Expected probability**	**Outcome**
Hematuria	2 in 3 men	Self-resolving, 3–14 days
Hematospermia	3 in 10 men	Self-resolving, 1–2 months
Bleeding in perineal	1 in 10 men	Self-resolving, 1–12 hours
Bleeding in rectum	1 in 50 men	Self-resolving, 1–3 days
Lower urinary tract symptoms	1 in 10 men	Self-resolving, 1–7 days
Transient erectile dysfunction	1 in 10 men	Self-resolving, 2–3 weeks
Urinary retention	1 in 30 men	Indwelling catheter, 3–7 days
Discomfort when passing urine	2 in 3 men	Self-resolving, 1–3 days
Discomfort in the rectum	2 in 3 men	Self-resolving, 1–3 days
Urinary tract infection	1 in 100 men	Oral antibiotics, 3–7 days
Fever	None	Intravenous antibiotics 3–7 days
Vasovagal event	1 in 50 men	Self-resolving, 0.5–2 h
Anaesthetic allergy	None	Antiallergy treatment, 0.5–3 h

### Blinding

All participants and research nurses measuring or recording the results will be unaware of the intervention group (perineal nerve block or periprostatic block). The researcher who collects or enters the data will also be blinded to the allocation. The urologist who conducts the anaesthesia will be blinded to all other data, including the name and trial number of the participant, and will not aid in assessing the outcomes or processing the data. The other urologist who will perform the prostate biopsy will be unaware of the allocation, and the pathologist who will report the pathologic outcomes will be unaware of all patients' clinical information.

### Data Collection and Monitoring

**Figure 4:**
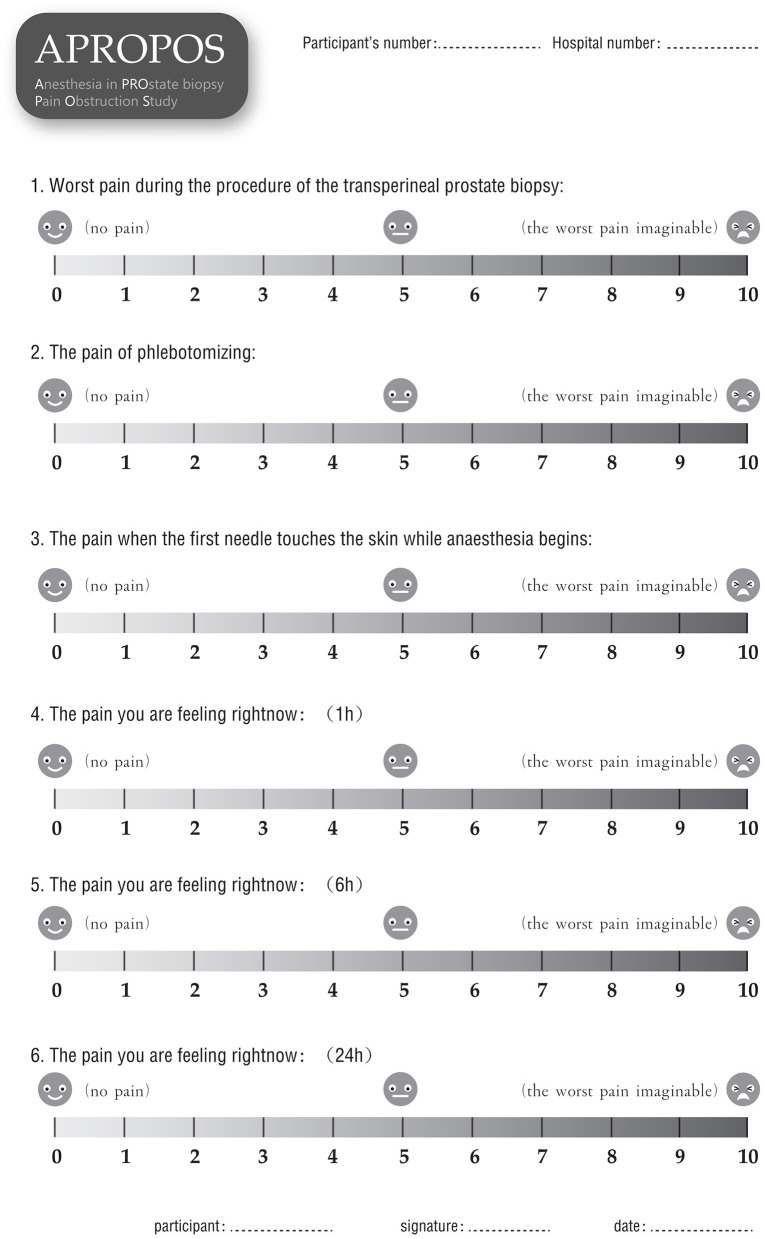
Form 1.

**Figure 5:**
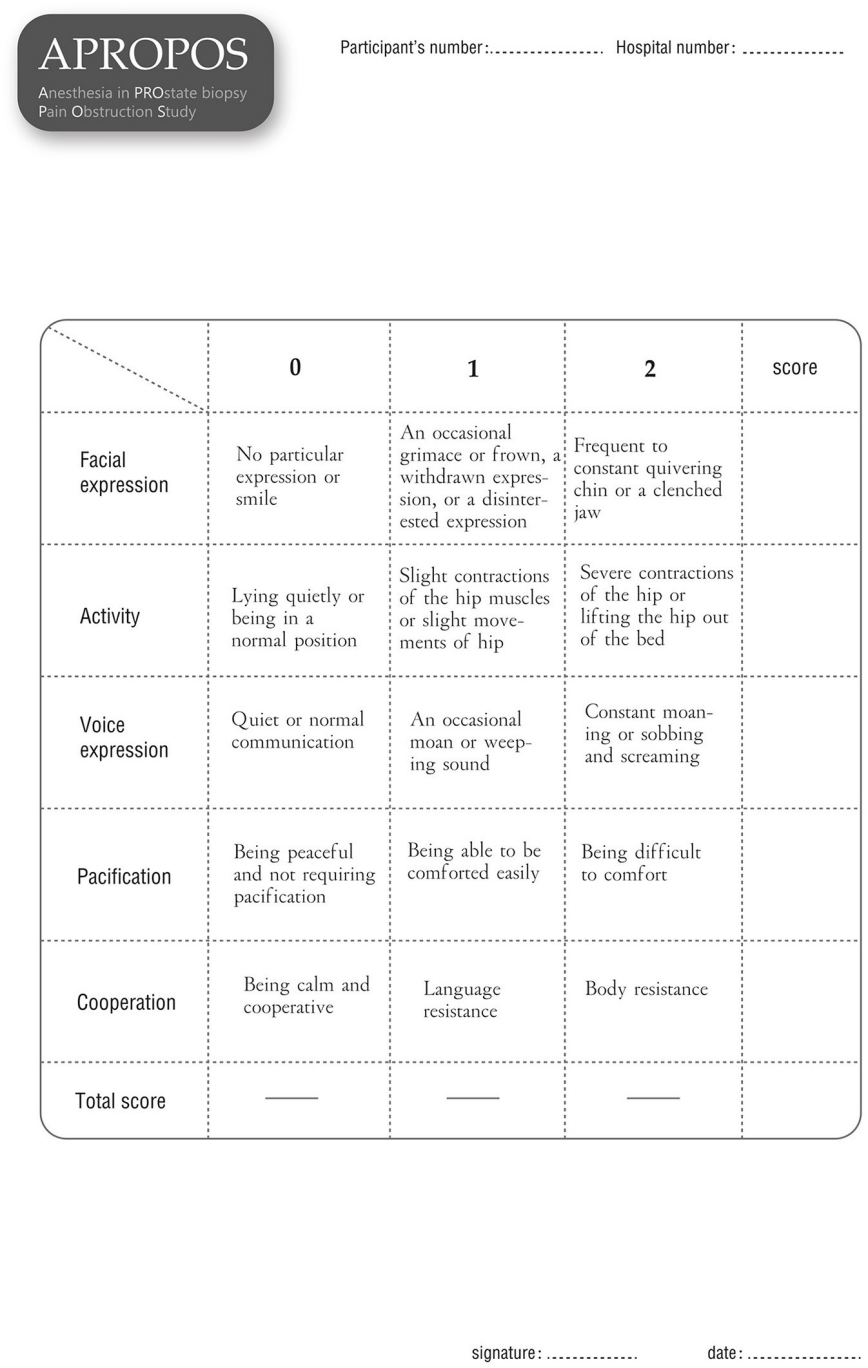
Form 2.

**Figure 6:**
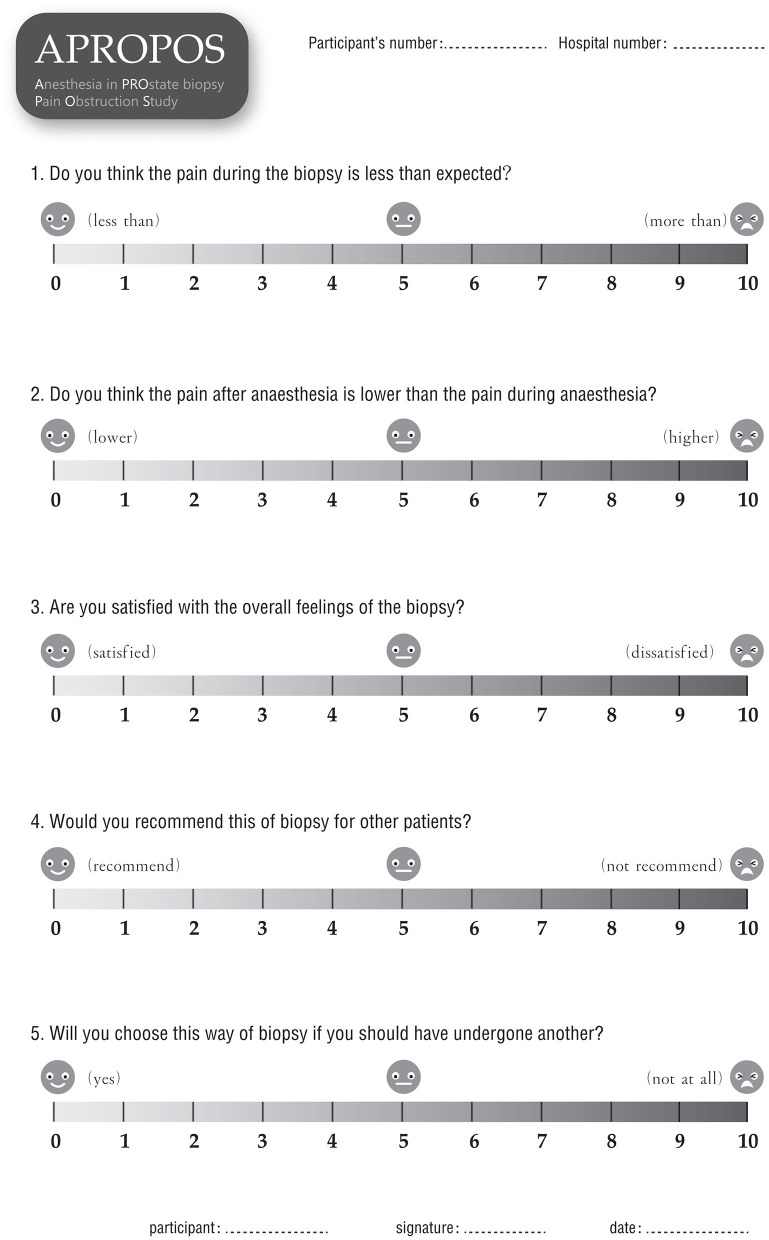
Form 3.

After informed consent is obtained from the patients, the data will be collected by a research nurse who is blinded to the allocation to ensure the integrity of evaluation and prevent bias. Demographic information, including age, body mass index (BMI), PSA level, DRE results, prostate volume and American Society of Anesthesiologists (ASA) classification will be collected before anaesthesia. The pain score will be measured and recorded *via* form 1 (Figure 4) at 10 min, 1, 6, and 24 h after the biopsy. Additionally, the pain of phlebotomizing and the pain at the first moment the needle touches the skin during anaesthesia will be used as the reference and baseline for the pain assessment (form 1). During the biopsy, the external manifestations of pain will be measured *via* form 2 (Figure 5), and the blood pressure, heart rate and breathing rate will be recorded by a multi-parameter monitor. The site of needle core will be reported, because the targeted cores of the anterior zone might be associated with more pain in comparison with those of the peripheric zone (19). Before the participant is dismissed, the anaesthesia satisfaction assessment will be collected *via* form 3 (Figure 6). The pathologic data will be collected within 2 weeks after the biopsy. All of these data will be entered into a particular database. An independent monitor from the APROPOS operation group will check the quality of the data at least twice a week. The monitor may pose queries to the data, and the validity of the data will not be confirmed unless the questions have been resolved.

## Discussion

We initially developed the perineal nerve block based on an anatomical study and then conducted a pilot trial to verify its efficacy and safety preliminarily.

To confirm the findings and ensure the results more generalizable, we plan to conduct this multicentre randomised controlled trial, used rigorous randomised design and blinding method.

Apart from the subjective NRS pain scores reported by the patients, the external manifestations of pain during the biopsy, and the indicator changes displayed on the monitor will be considered to make the pain control assessment more comprehensive and multidimensional.

In conclusion, this trial will determine the efficacy of the perineal nerve block in controlling pain in patients undergoing prostate biopsy *via* the transperineal approach.

## Trial Status

This RCT was first registered online at ClinicalTrials.gov on August 2, 2020. The trial is start recruiting on August 13, 2020. Recruitment is anticipated to continue until September 15, 2021, with the 1-month follow-up expected to be completed on October 15, 2021.

## Ethics Statement

Ethical approval was obtained from the Ethics Committee of all centres (Supplementary Material). All participants will sign a consent form prior to randomisation. The results of this trial will be disseminated to an international peer-reviewed journal and presentations at international or national academic conferences.

## Patient and Public Involvement

The patients and public were not involved in the contribution of the design, recruitment or conduction of the trial. Each participant will be informed of the latest results at follow-up and received a summary of the main finding at the end of the trial.

## Author Contributions

Study protocol was conceived and designed by B-MH and H-FW and revised critically by R-BL. The original draft was completed by B-MH. All authors participated in the editing of the protocol.

## Funding

This trial was supported by National Key Research and Development Program (2019YFC0119100) from National Natural Science Foundation of China and supported by Shanghai Science and Technology Commission Foundation (18441910900) from Science and Technology Commission of Shanghai Municipality.

## Conflict of Interest

The authors declare that the research was conducted in the absence of any commercial or financial relationships that could be construed as a potential conflict of interest.

## Publisher's Note

All claims expressed in this article are solely those of the authors and do not necessarily represent those of their affiliated organizations, or those of the publisher, the editors and the reviewers. Any product that may be evaluated in this article, or claim that may be made by its manufacturer, is not guaranteed or endorsed by the publisher.
