# Supplementary material for: Anaesthesia in PROstate Biopsy Pain Obstruction Study: A Study Protocol for a Multicentre Randomised Controlled Study Evaluating the Efficacy of Perineal Nerve Block in Controlling Pain in Patients Undergoing Transperineal Prostate Biopsy
Source: Front Surg. 2021 Oct 6;8:649822. doi: 10.3389/fsurg.2021.649822 (PMC8527031; doi:10.3389/fsurg.2021.649822)
Supplement: Supplementary file 1 [file Data_Sheet_1.PDF]

Table supplement. The APROPOS Group

Member	Centre	Ethics Board	Region
Dr Bi-Ming He	Department of Urology, Shanghai East Hospital, Tongji University School of Medicine	shanghai east hospital ethics committee	Shanghai, China
Dr Rong-Bing Li	Department of Urology, Shanghai East Hospital, Tongji University School of Medicine	shanghai east hospital ethics committee	Shanghai, China
Dr Hai-Feng Wang	Department of Urology, Shanghai East Hospital, Tongji University School of Medicine	shanghai east hospital ethics committee	Shanghai, China
Dr Qing-song Yang	Department of Radiology, Changhai Hospital, Second military medical university	Changhai hospital ethics committee	Shanghai, China
Dr Jun Xiao	Department of Urology, The affiliated hospital of USTC,University of Science and Technology of China	The affiliated hospital of USTC ethics committee	Hefei, China
Dr Guang-Yuan Li	Department of Urology, The Fourth Affiliated Hospital of Anhui Medical University	The Fourth Affiliated Hospital of Anhui Medical University ethics committee	Hefei, China
Dr Ben-Kang Shi	Department of Urology, Qilu hospital of Shandong university, Shandong University	Qilu hospital ethics committee	Jinan, China
Dr Li-Cheng Wu	Department of Urology,Tongji Hospital, Tongji Medical College, Huazhong University of Science and Technology	Tongji hospital ethics committee	Wuhan, China
Dr Jian-Bin Bi	Department of Urology, The first hospital of China Medical University	The first hospital of China Medical University ethics committee	Shenyang, China
Dr Bin Hu	Department of Urology, Liaoning Cancer hospital	Liaoning Cancer hospital ethics committee	Shenyang, China
Dr Wan-Hai Xu	Department of Urology, The 4th Affiliated Hospital of Harbin Medical University	The 4th Affiliated Hospital of Harbin Medical University ethics committee	Harbin, China
Dr Yi-Fei Liu	Department of Urology, Tangshan center hospital	Tangshan center hospital ethics committee	Tangshan, China
Dr Lie-Fu Ye	Department of Urology, Fujian provincial hospital	Fujian provincial hospital ethics committee	Fuzhou, China
Dr Guo-lin Lei	Department of Urology, The people's hospital of Jianyang city	The people's hospital of Jianyang city ethics committee	Jianyang, China
Dr Jian-Hua Lan	Department of Urology, West China - Guang'an hospital, Sichuan university	Guang'an hospital hospital ethics committee	Guang'an, China
Dr Ying-Li Yang	Department of Urology, Cangxi people's hospital	Cangxi people's hospital ethics committee	Cangxi, China
Dr Wen Luo	Department of Urology, Gulin people's hospital	Gulin people's hospital ethics committee	Gulin, China
Dr Jian-Tong Cai	Department of Urology, Shishi Hospital	Shishi hospital ethics committee	Shishi, China
Dr Xu-Lai Tao	Department of Urology,Yiyuan Hospital	Yiyuan hospital ethics committee	Yiyuan, China
